# 

**Published:** 1876-01

**Authors:** 


### Table of Contents.

### ORIGINAL COMMUNICATIONS.

Immobility or Closure of the Jaw. *By W. F. Westmoreland, M.D.* .... 577
Dislocation of an Eye-ball. *By A. W. Calhoun, M.D.* ........ 587

### CLINIC ATLANTA MEDICAL COLLEGE.

Service of J. G. Westmoreland, M.D. ........ 590
Service of W. F. Westmoreland, M.D. ........ 596
Eye and Ear, A. W. Calhoun, M.D. ........ 600

### ATLANTA ACADEMY OF MEDICINE.

Opium Antidote—Uræmic Poisoning—Puerperal Convulsions—Blood Letting—Operation for Cancer—Diphtheria........ 603

### OUR EXCHANGES.

On the Sequelæ and Prophylaxis of Simple Tonsillar Hypertrophy in Children........ 610
Artificial Dilatation of the Cervix in Obstinate Vomiting of Pregnancy.. 615
Acute Albuminuria of Pregnancy ........ 616
A Novel Method of Treating the Vomiting of Pregnancy........ 617
Living Larvæ in the Ear, and Methods for their Destruction........ 619
The "Multiple Wedge Principle" Applied to the Continuous Method of Dilatation of Stricture of the Urethra........ 622
Nervous Disorders from Genital Irritation ........ 623
Monstrosity by Inclusion ........ 625
Removal of Tape-Worm by Balsam Copaiba ........ 626
Nitrite of Amyl in Nervous Cephalalgia. ........ 627
Post-Partum Pill ........ 627
Pruritus Treated with Vinegar........ 627
The Value of Belladonna as a Prophylactic in Scarlet Fever........ 628
New Method of Treatment of Febrile Articular Rheumatism........ 628
Hypodermic Use of Carbolic Acid in Rheumatism........ 629
Eczema Produced by Tincture of Arnica........ 629
The Catheter in Enlarged Prostate........ 629
Sulphuric Acid in the Treatment of Boils........ 630
Formula for Combined Administration of Cod-Liver Oil and Phosphorus 630

### BIBLIOGRAPHICAL.

On Poisons in Relation to Medical Jurisprudence and Medicine........ 631
Transactions of the College of Physicians of Philadelphia........ 631
Cyclopædia of the Practice of Medic ne........ 631
Hospital Construction and Organization........ 632
Transactions of Illinois State Medical Society........ 632
Hints on the Obstetric Practice ........ 633
The Louisville Medical News........ 633
Pamphlets........ 633

### EDITORIAL.

Greeting........ 634
Errata........ 635
On Dit. ........ 635
International Medical Congress........ 638

